# Acute Noninfectious Anterior Ocular Inflammation Following Ranibizumab Biosimilar Intravitreal Injection in a Patient With Recent COVID-19 Vaccination

**DOI:** 10.7759/cureus.60356

**Published:** 2024-05-15

**Authors:** Ryo Tetsumoto, Wataru Matsumiya, Rei Sotani, Sentaro Kusuhara, Makoto Nakamura

**Affiliations:** 1 Division of Ophthalmology, Department of Surgery, Kobe University Graduate School of Medicine, Kobe, JPN

**Keywords:** ranibizumab biosimilar, vau, uveitis, intravitreal injection, ocular inflammation, covid-19 vaccination

## Abstract

Even in the post-coronavirus disease 2019 (COVID-19) era, it is prudent to exercise caution regarding the timing between intravitreal anti-vascular endothelial growth factor (VEGF) injections and COVID-19 vaccinations, as ocular inflammation can occur following both procedures. However, this perspective has not been sufficiently discussed thus far. Herein, we report a case of acute noninfectious anterior ocular inflammation following an intravitreal injection of ranibizumab biosimilar (RBZ BS, Senju Pharmaceuticals, Japan) in a patient recently vaccinated against COVID-19. A 74-year-old male with myopic choroidal neovascularization (CNV) in the left eye was treated with RBZ BS intravitreal injection. He received his fourth COVID-19 vaccination with messenger ribonucleic acid (mRNA)-1273 (Moderna) two days prior to his second RBZ BS intravitreal injection. He reported no systemic symptoms associated with the fourth COVID-19 vaccination. The second RBZ BS intravitreal injection was safely performed without complications. However, a few hours later, he experienced blurred vision without ocular pain in his left eye, a symptom not observed after the first injection. He visited a local ophthalmologic clinic the following day and was subsequently referred to our hospital due to anterior ocular inflammation in the left eye. His vision in the left eye was 0.3 decimal best-corrected visual acuity. Examination revealed non-granulomatous anterior ocular inflammation with 3+ cells and 2+ flare in the left eye. Anterior vitreous inflammation, keratic precipitates, or conjunctivitis was absent. Fundus examination also showed no signs of posterior inflammation. Both fluorescence angiography and indocyanine green angiography revealed staining corresponding to CNV without retinal vasculature leakage. There is nothing abnormal with the right eye based on the examination. Given that the noninfectious ocular inflammation was likely, based on the acute onset of symptoms within less than 24 hours following the RBZ BS intravitreal injection, and the presence of non-granulomatous inflammation only in the anterior segment without ocular pain, betamethasone eye drops four times daily was initiated in the left eye on the first day following the second RBZ BS intravitreal injection. Then, his ocular inflammation improved to mild by the fourth day post-injection. His eye eventually cleared, with no cells or flare in the anterior chamber at five months. Eventually, given the clinical course of good response to only topical steroid therapy, the diagnosis of noninfectious anterior ocular inflammation following RBZ BS in the case of a recent episode of COVID-19 vaccination was retrospectively confirmed. Although this case represents one of the initial instances of noninfectious ocular inflammation following RBZ BS (Senju Pharmaceuticals) administration, sterile ocular inflammation after other intravitreal anti-VEGF therapy has already been well-reported. In addition, given the recent COVID-19 vaccination, the ocular inflammation might be influenced by the vaccination, synergistically leading to vaccine-associated uveitis with similar signs and symptoms. In conclusion, to prevent such a complex situation, it is advisable to consider an adequate interval between COVID-19 vaccination and intravitreal anti-VEGF injections.

## Introduction

Intravitreal injection of anti-vascular endothelial growth factor (VEGF) agents is the gold standard therapy for myopic choroidal neovascularization (CNV), as well as for neovascular age-related macular degeneration (AMD) and diabetic macular edema [1]. These drugs need to be administered repeatedly to achieve and maintain clinical benefits. Therefore, biosimilars of anti-VEGF agents have been recently approved for clinical use to reduce economic burden. On the other hand, despite their known safety and tolerability, physicians must remain vigilant for any side effects following each intravitreal injection of anti-VEGF agents including biosimilars, even if they are infrequent [2]. Furthermore, acute noninfectious intraocular inflammation following intravitreal anti-VEGF agent injections has sometimes been reported, although it is rare [3].

After the coronavirus disease 2019 (COVID-19) pandemic, messenger ribonucleic acid (mRNA) vaccine technology for COVID-19 was widely adopted around the world, becoming a common form of vaccination [4]. The mRNA-1273 (Moderna) vaccines are representative examples of mRNA COVID-19 vaccines as well as BNT162b2 (Pfizer-BioNTech). Since their introduction, cases of COVID-19 vaccine-associated uveitis (VAU) have been reported as rare ocular side effects of the COVID-19 vaccines. Although VAU can manifest as various types of ocular inflammation, anterior uveitis is the most common form of VAU [5]. In the post-COVID-19 pandemic era, COVID-19 VAU should be considered an important differential diagnosis for patients presenting with uveitis, especially those with a recent history of COVID-19 vaccination.

In general, when ocular inflammation follows an intravitreal anti-VEGF agent injection in clinical practice, it is crucial to differentiate between infectious and noninfectious ocular inflammation to administer appropriate treatment and preserve vision. However, as differentiating the cause of ocular inflammation can be challenging, the underlying cause is sometimes elucidated through a retrospective review of the case [6]. Furthermore, a recent history of COVID-19 vaccination complicates the accurate diagnosis of ocular inflammation after the intravitreal injection. In this context, we report a case of anterior ocular inflammation following intravitreal ranibizumab biosimilar (RBZ BS, Senju Pharmaceuticals, Japan) occurring after a recent COVID-19 vaccination episode.

## Case presentation

**Figure 1:**
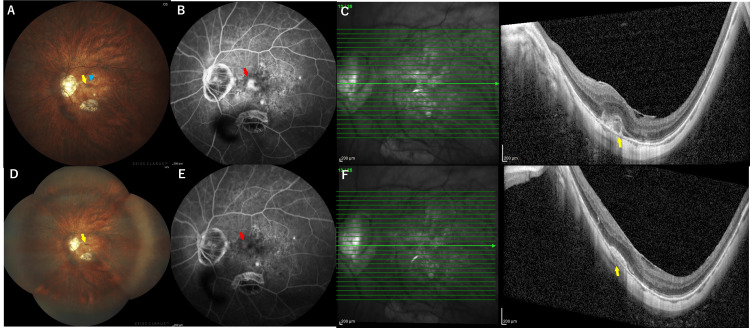
Clinical examinations including the fundus photo, FA, and OCT in the OS. (A) At the initial presentation, the fundus of OS highlighted the gray-white foveal lesion (yellow arrow) accompanied by subretinal hemorrhage (blue arrowhead). The fundus also displays myopic changes, such as localized chorioretinal atrophy, posterior staphyloma, and a tilted disc with conus. (B) Late-phase FA revealed substantial foveal leakage (a red arrow) in OS, exhibiting a classic CNV pattern with no signs of ocular inflammation. This finding led to the diagnosis of myopic CNV in the OS. (C) SHRM with SRF at the fovea (yellow arrow) was observed on OCT. (D) On day 1 following the second ranibizumab BS intravitreal injection, the fundus showed a small gray-white foveal lesion (yellow arrow) but no evidence of vasculitis or posterior inflammation, despite the presence of anterior ocular inflammation. (E) Late-phase FA revealed just staining corresponding to CNV (a red arrow) without any leakage from the retinal vasculature. (F) OCT showed flattened SHRM without SRF (yellow arrow) in the OS. OS: left eye; FA: fluorescein angiography; CNV: choroidal neovascularization; SHRM: subretinal hyperreflective material; SRF: subretinal fluid; OCT: optical coherence tomography

A 74-year-old male presented to our hospital with central scotoma in the left eye (OS). His medical history included cataract surgery OS two months prior and his third COVID-19 vaccination four months ago. He denied any history of uveitis or any systemic diseases other than hypertension. Upon initial examination, the anterior segment was quiet in both eyes (OU). He had a grade 2 cataract based on the Emery-Little classification in the right eye (OD) and an intraocular lens in the OS. Examination of the posterior segment revealed myopic fundus changes OU, including localized chorioretinal atrophy, posterior staphyloma, and a tilted disc with conus. Notably, the left eye exhibited a gray-white lesion with subretinal hemorrhage in the parafoveal area, and subretinal hyperreflective material (SHRM) with subretinal fluid at the fovea was observed on optical coherence tomography (OCT) (Figure 1A-1C).

At that visit, his decimal best-corrected visual acuity (BCVA) was recorded as 0.6 in the OD and 0.3 in the OS. He had pathological myopia, with an axial length of 30.6 mm OU. Fluorescein angiography (FA) revealed significant leakage at the fovea in the OS, displaying a classic CNV pattern without any evidence of ocular inflammation, leading to a diagnosis of myopic CNV in the OS.

On the same day, an intravitreal injection of RBZ BS was administered in the OS without any complications. To ensure sanitization, both the physician and the patient wore masks. Prior to the intravitreal injection, the eyelid was cleansed with a 10% povidone-iodine (PI) swab, followed by a wash of the ocular surface with a 5% PI solution. A 0.5% moxifloxacin hydrochloride eye drop was also routinely applied for four days post-injection.

One month later, SHRM observed on OCT had almost improved, and the parafoveal grayish lesion in the OS had diminished. Consequently, a second intravitreal injection of RBZ BS was safely performed without any complications. Notably, in the context of the COVID-19 pandemic era in 2022 in Japan, he received his fourth COVID-19 vaccination two days before this second RBZ BS injection. He reported no systemic adverse effects following the fourth COVID-19 vaccination.

**Figure 2:**
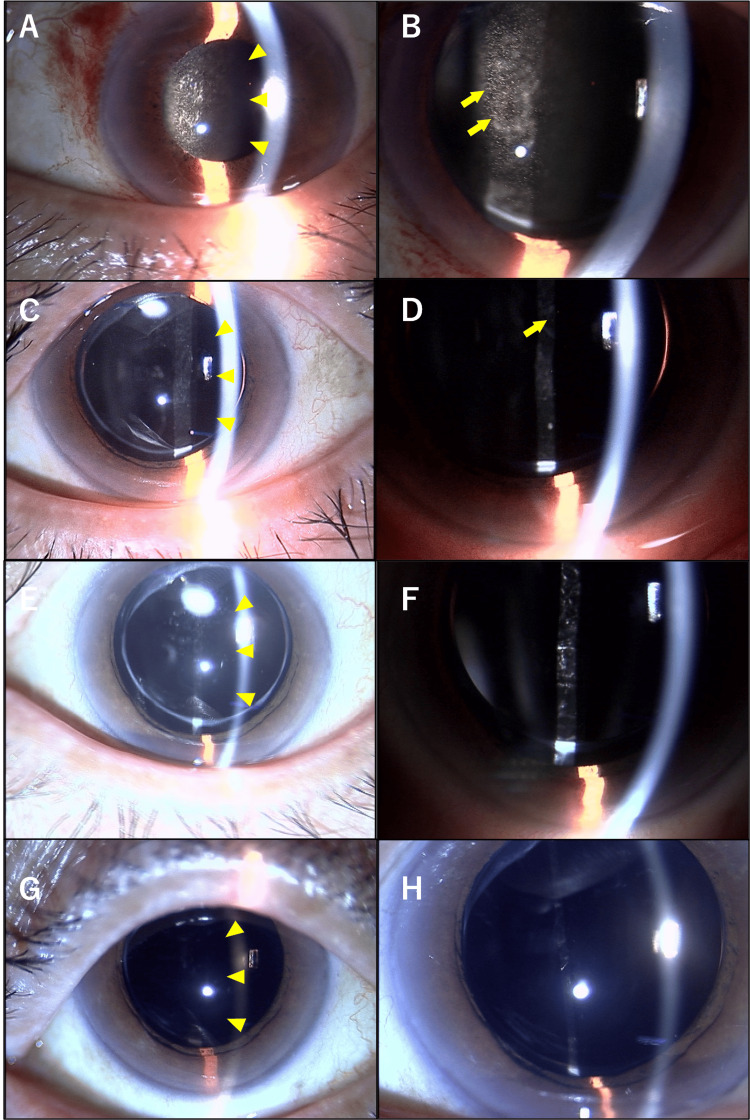
Clinical course of the anterior segment in the OS. The images labeled A, C, E, and G depict the comprehensive condition of the anterior segment, including the conjunctiva, cornea, iris, and intraocular lens, in the OS. The images labeled B, D, F, and H provide a closer view of the anterior chamber by enlarging the details from images A, B, C, and D, respectively, to focus on the changes occurring within the anterior chamber over time. (A, B) On day 1 following the second ranibizumab BS intravitreal injection, non-granulomatous anterior ocular inflammation with 3+ cells (yellow arrows) and 2+ flare (yellow arrowheads) was observed in the OS. There was neither fibrin nor hypopyon in the OD. The logMAR BCVA in the OS was 0.52. (C, D) On day 4, after three days of using 0.1% betamethasone eye drops four times daily, the ocular inflammation in the OS had diminished to mild anterior ocular inflammation with 1+ cell (yellow arrows)  and 1+ flare (yellow arrowheads). The logMAR BCVA in the OS improved to 0.30. (E, F) At six weeks, the anterior segment in the OS showed minimal ocular inflammation with 0.5+ cells and no flare (yellow arrowheads). The logMAR BCVA in the OS was 0.40. (G, H) At five months, the anterior segment in the OS was completely clear with no cells and no flare (yellow arrowheads). The logMAR BCVA in the OS remained stable at 0.40. OS: left eye; OD: right eye; BCVA: best-corrected visual acuity

A few hours later the day of the RBZ BS injection, he noticed blurred vision in the OS. Aware of this new symptom, which was not observed after the first injection, he visited a local ophthalmologic clinic the next day. He reported no ocular pain in the OS. Eventually, he was referred to our hospital due to anterior ocular inflammation in the OS. Examination revealed non-granulomatous anterior ocular inflammation with 3+ cells and 2+ flare, without any inflammation in the anterior vitreous. The cornea was clear, with no keratic precipitates (KPs) and no signs of conjunctivitis (Figure 2A, 2B). 

The OD was unremarkable. His vision remained at 0.3 decimal BCVA in the OS. The fundus examination showed no signs of vasculitis or posterior inflammation. FA revealed staining corresponding to CNV without leakage from the retinal vasculature (Figure 1D-1F). Although infectious endophthalmitis was initially considered, the clinical presentation, characterized by immediate onset, exclusive anterior ocular inflammation with no pain, and non-granulomatous inflammation without fibrin or hypopyon, indicated that it was likely to be noninfectious ocular inflammation. In addition, despite undergoing a comprehensive systemic review, the patient reported no symptoms indicative of collagen or autoimmune diseases, and based on the results of laboratory tests, infectious etiologies such as tuberculosis, syphilis, and toxoplasmosis were denied. Consequently, the patient started treatment for noninfectious anterior uveitis following an injection of RBZ BS, occurring after a recent fourth COVID-19 vaccination episode.

After initiating treatment with 0.1% betamethasone eye drops four times daily on the first day following the second ranibizumab intravitreal injection, his ocular inflammation improved to mild anterior inflammation by day 4 post-injection. Four days after the second RBZ BS injection, the anterior chamber in the OS showed 1+ cells and 1+ flare (Figure 2C, 2D). Six weeks later, the anterior chamber in the OS exhibited 0.5+ cells and no flare (Figure 2E, 2F). Subsequently, after tapering off 0.1% betamethasone, the treatment was switched to 0.1% fluorometholone eye drops twice daily.

Two months later, as myopic CNV recurred in the OS, he received another additional intravitreal anti-VEGF injection, aflibercept, for the myopic CNV. Following this treatment, he experienced no further ocular inflammation.

At five months post-injection, the anterior chamber in the OS was completely quiet, with no cells and no flare. He then discontinued the steroid eye drops, and his vision was recorded as 0.4 decimal BCVA in the OS (Figure 2G, 2H).

## Discussion

Ocular inflammation associated with intravitreal RBZ BS

RBZ BS (Senju Pharmaceuticals, Japan) was the first biosimilar of an anti-VEGF drug for ocular diseases in Japan. A phase 3 randomized clinical trial involving 351 subjects with wet AMD in Japan demonstrated the equivalence of RBZ BS to ranibizumab (Novartis Pharma), confirming its long-term safety and efficacy [7]. Due to the lower price of RBZ BS compared to other anti-VEGF drugs, it is anticipated that RBZ BS will become the preferred treatment for patients with neovascular AMD in treat-and-extend and pro re nata regimens as well as the best supportive care plan.

Large-scale studies have reported the frequency of infectious endophthalmitis associated with original anti-VEGF agents as an adverse event to be between 0.02% and 0.14%, while the incidence of sterile endophthalmitis after intravitreal anti-VEGF therapy ranges from 0.005% to 4.4% [3]. Notably, severe ocular inflammation was reported to occur with ranibizumab, at 0.64 per 1000 injections, which is less than with aflibercept, at 1.06 per 1000 injections [8]. Regarding ocular adverse events of RBZ BS, which was approved as a biosimilar of ranibizumab in September 2021 for the treatment of CNV in pathologic myopia in Japan, a trial reported no cases of iritis or intraocular inflammation out of eight eyes with ocular adverse effects (4.57%) [9]. Additionally, post-market surveillance conducted by Senju Pharmaceuticals has not reported any noninfectious intraocular inflammation following RBZ BS administration. Therefore, the present case is one among the initial instances of noninfectious ocular inflammation following RBZ BS administration.

Anderson et al. proposed three main causes of sterile intraocular inflammation after anti-VEGF injection: patient-specific, medication-specific, and delivery-specific. Regarding the medication-specific aspect, one of the plausible hypotheses is that extrinsic epitopes present on the anti-VEGF molecule might be recognized by antigen-presenting cells and subsequently presented to B cells and T cells. This interaction could trigger the production of antibodies and the generation of memory cells, leading to an enhanced immune reaction [10]. Therefore, anti-VEGF injections, whether biosimilar or not, can lead to sterile ocular inflammation, although such occurrences are quite rare.

Differential diagnosis of anterior intraocular inflammation following the intravitreal injection of RBZ BS

Our institution has rigorously adhered to the protocols outlined in the Japanese Guidelines for Intravitreal Injection for Macular Diseases. These protocols mandate that both physicians and patients wear masks, utilize topical PI solution during intravitreal injections, and apply topical antibiotics for four days post-procedure [11].

Nonetheless, endophthalmitis remains the most critical differential diagnosis to consider. In this particular case, the patient experienced blurred vision without eye pain a few hours following the intravitreal injection of RBZ BS and was diagnosed with anterior uveitis the day after the injection. The sudden onset within 24 hours post-RBZ BS injection, the absence of ocular pain, and negative evidence of conjunctivitis, KPs, fibrin, and hypopyon suggested a noninfectious intraocular inflammation rather than endophthalmitis [3]. The ocular inflammation was also confined to the anterior segment without extending to the vitreous or posterior segment [6]. Eventually, the diagnosis of noninfectious ocular inflammation following intravitreal injection was also retrospectively confirmed based on the clinical course, as the intraocular inflammation following RBZ BS completely resolved with only steroid eye drop treatment.

A comprehensive systemic analysis, including blood and urine tests, revealed no indicators of systemic diseases related to anterior uveitis. Conditions such as diabetes mellitus, ocular tuberculosis, ocular syphilis, and other autoimmune diseases were ruled out through blood tests. The patient also denied experiencing specific symptoms associated with Behçet's disease or spondylarthritis, such as oral ulcers, skin rash, genital ulcers, back pain, or joint pain. Thus, the current case is likely attributable to noninfectious ocular inflammation related to RBZ BS.

Possible impact of COVID-19 vaccination

In the current case, the fourth COVID-19 vaccination, using the mRNA-1273 vaccine, was administered two days before the onset of ocular inflammation. Although VAU had been reported before the COVID-19 era [12], there has been a notable increase in reports of ocular inflammation following the administration of COVID-19 vaccines, particularly mRNA vaccines. The most common type of ocular inflammation is anterior uveitis, which accounts for more than half of all cases of COVID-19 VAU [13]. Regarding the frequency and timing of COVID-19 VAU, noninfectious ocular inflammation has been reported at rates of 66.8 and 62.7 cases per 100,000 person-years following the first and second doses of the BNT162b2 mRNA vaccine, respectively [5]. Out of a total of 1094 cases of VAU reported from 40 countries, the observed-to-expected ratio of VAU was comparable for BNT162b2 (0.023) and mRNA-1273 (0.025), with most cases reported within the first week (n=591, 54.02%) of vaccination [14,15]. Analysis based on the Centers for Disease Control and Prevention (CDC) Vaccine Adverse Event Reporting System (VAERS) database also indicated that the median onset duration for VAU was four days [14]. Thus, in this case, anterior uveitis might be also associated with a history of COVID-19 vaccination which was administrated two days prior to RBZ BS injection.

There have been a few reports on the association between inflammation after surgical stress and COVID-19 vaccination. Dermatologists have cautioned about the possibility of transient inflammation in surgical scars following COVID-19 mRNA vaccination [16]. Additionally, a case of Wolf's isotopic response following COVID-19 vaccination (MVC-COV1901), which describes the occurrence of a new skin disorder at the site of another unrelated and already healed skin lesion, has been recently reported [17].

In the case at hand, determining the exact cause of the transient severe anterior uveitis is challenging. Without evidence of a systemic disorder, it's conceivable that the RBZ BS intravitreal injection, the recent episode of COVID-19 vaccination, or a combination of both could have precipitated the ocular inflammation. Additionally, the recent COVID-19 vaccination might have synergistically triggered the onset of ocular inflammation following RBZ BS administration.

Regarding the mechanism of COVID-19 VAU, previous reports have highlighted the role of vaccine-induced interferon type I (IFN-I) in the interaction between mRNA COVID-19 vaccination and VAU [18]. The mRNA vaccines activate IFN-I production through RNA sensors, including retinoic acid-inducible gene-I (RIG-I). This activation promotes the differentiation of cluster of differentiation 4+ (CD4+) and CD8+ effector T cells, which are associated with inflammatory and cytotoxic mediators. Additionally, CD4+ helper T cells are stimulated, promoting the differentiation of B cells into plasma cells capable of secreting antibodies [18]. Given the proposed mechanism of sterile ocular inflammation following anti-VEGF intravitreal injections, COVID-19 vaccination might have not only caused ocular inflammation but also had a synergistic effect with RBZ BS, resulting in ocular inflammation in the present case. Regarding the synergistic side effects between the COVID-19 vaccine and medications, there have been reports highlighting the potential for the onset of bullous pemphigoid when taking dipeptidyl peptidase 4 inhibitors (DPP4-i) after COVID-19 vaccination [19]. However, a synergistic ocular side effect between the COVID-19 vaccine and intravitreal anti-VEGF agents has not yet been reported.

Consequently, considering the risk of ocular inflammation, particularly endophthalmitis following intravitreal injection, it is prudent to be cautious about the timing between intravitreal injections and COVID-19 vaccinations. Most existing guidelines recommend a separation of one to two weeks between COVID-19 vaccination and general surgery [20]. Therefore, to mitigate the risk of VAU as an adverse effect of COVID-19 vaccination, it is advisable for patients slated for anti-VEGF intravitreal injections to have completed their COVID-19 vaccination at least two weeks prior.

## Conclusions

This case represents one of the initial instances of noninfectious ocular inflammation following RBZ BS (Senju Pharmaceuticals) administration. In addition, the recent episode of COVID-19 vaccination could influence the onset or severity of ocular inflammation. Therefore, this case underscores the importance of determining an appropriate interval between COVID-19 vaccination and anti-VEGF intravitreal injections to minimize potential complications.
